# Perceived quality of life and life style modification of cancer patients undergoing varied treatments in a tertiary health institution, Ekiti State, Nigeria

**DOI:** 10.11604/pamj.2021.40.227.27454

**Published:** 2021-12-16

**Authors:** Deborah Tolulope Esan, Khadijat Toyin Musah, Fayokemi Mary Olaiya, Opeyemi Adeniyi Adedeji, Emmanuel Omoniyi Olowolafe

**Affiliations:** 1 Department of Nursing Science, College of Medicine and Health Sciences, Afe Babalola University, Ado-Ekiti, Nigeria,; 2 Department of Public Health, School of Basic Medical Sciences, Community Health Unit, Kwara State University, Malete, Nigeria,; 3 Obstetrics and Gynecology Department, University College Hospital, Ibadan, Nigeria

**Keywords:** Cancer patients, quality of life, lifestyle modification, Nigeria, African context

## Abstract

**Introduction:**

cancer is viewed in the African context as a death sentence. Its effect can be overwhelming to both the patient and their support system. The objective of the study was to assess the perceived quality of life of cancer patients undergoing varied cancer treatments in a tertiary health institution in Ekiti State and to determine the life style modification of cancer patients undergoing varied treatments in the same health facility.

**Methods:**

the study employed a descriptive cross-sectional design. A consecutive sampling approach was utilized to select 80 respondents among the cancer patients who attended the cancer registry within the study time frame. Data was collected from these cancer patients by using structured and validated questionnaire. Data was analyzed using descriptive statistics and inferential statistics with level of significance set at p < 0.05.

**Results:**

respondent´s ages ranged from 20 - 61 years with a mean age of 50 ± 18.3 years. Only 7.8% are not dependent on medications to function in their daily life. Most of the participants reported that their sex life has been affected (61.1%) and 77% of the respondents reported fatigue. About 76% of respondents need varying measure of medical treatment to function in their daily life. Overall, 44.8% have poor quality of life, while 55.1% had good quality of life in this current study. Moreover, a significant relationship was found between quality of life and self-assessment of patient´s health (p < 0.001). Multivariate analysis predicting factors affecting quality of life of respondents revealed that self-assessment of respondents (AOR: 3.389; 95% CI: 1.897-6.054) remained a significant and more likely predictor of quality of life while respondent´s age (AOR: 0.244; 95% CI: 0.068-0.876) and level of education (AOR: 0.054; 95% CI: 0.005-0.546) were less likely predictors.

**Conclusion:**

one quarter of the participants have poor life and majority of the participants need varying measure of medical treatment to function in their day to day life. Management of cancer patients should be geared towards improving/ameliorating symptoms and improving quality of life of cancer patients.

## Introduction

Cancer is one of the most life threatening illness that may appear at any point in a person´s life. Globally, more than 7 million people lose their lives from cancer and it is expected that the number of new cases will increase from 10 million to 15 million per year by 2020 [1,2]. Cancer is bothersome and gaining momentum fast in Nigeria and other developing countries. Despite, significant advances in medical sciences, cancers are discussed as one of the most important diseases as of today [1,2]. In society like ours (Nigeria), cancer is known as incurable disease and after diagnosis; patients suffer from anxiety and depression resulting from unrealistic fear of death and loss of happiness as the need for frequent hospitalization and continual concern for patients and their families and cause psychological disorders. When cure is impossible, often as a result of patients reporting to the hospital very late when the disease had already advance, goals change from intent to cure to the prolongation of life and palliation of symptoms. During this stage of disease, quality-of-life issues are particularly important [3].

Quality of life (QOL) is an important parameter to be considered when treating cancer patient. Quality of life, is the general well-being of individuals and societies, outlining negative and positive features of life. It observes life satisfaction, including everything from physical health, family, education, employment, wealth, safety, and security to freedom, religious beliefs, and the environment [4]. Gotay *et al*. [5] define QOL as the state of well-being that is combination of two components; the ability to perform everyday activities, social well-being and satisfaction with levels of functioning and control of the disease.

Ferrell and Dow have explained the domain for cancer survivors with four parameters, which include physical well-being, psychological well-being, social well-being and spiritual well-being [6]. Nowadays, people are demanding patients with cancer enter therapy with the recognition that therapy aimed at cure is often accompanied by side effects that have a negative impact on their quality of life. In recent years, many clinical cancer treatment research protocols have included a quality-of-life feature to evaluate the balance between side effects and quality of life during sometimes highly toxic treatment regimens. The study therefore assessed the perceived quality of life of cancer patients undergoing varied cancer treatments in a tertiary health institution in Ekiti State, determined the life style modification of cancer patients and also determined the factors affecting the quality of life of cancer patients.

## Methods

**Study design and setting:** the study adopted a descriptive cross-sectional design because the variables being studied had occurred and cannot be manipulated by the researcher. The study was conducted in Federal Teaching Hospital Ido-Ekiti, Ekiti State, Nigeria. The hospital is located in a suburban area in Ido-osi Local Government Area of Ekiti State, Nigeria. This government owned hospital is a 280-bed tertiary institution formerly known as Federal Medical Centre, Ido-Ekiti. The hospital serves is a referral centre for all other health institutions in Ekiti State. This hospital has 24 fully functioning departments comprising of 18 clinical departments. It has a capacity of 280 beds spanned through the following wards: male surgical, female surgical, male medical, female medical, paediatrics, accident and emergency, psychiatric, obstetrics and gynaecology and neonatal wards, surgical and medical outpatient´s department inclusive. In addition, it has a cancer registry, where cancer patients within and outside the state visit. It also has functional renal unit, cardiac unit, intensive care unit, ear, nose and throat and ophthalmology units.

**Study population:** the entire cancer patients in Federal Teaching Hospital, Ido-Ekiti, Ekiti State, Nigeria constituted the population of the study. The inclusion criteria included cancer patients: 1) Adults who have been diagnosed with cancer; 2) currently receiving treatment at Federal Teaching Hospital, Ido-Ekiti, and are attending the cancer registry of the hospital, as at the time of study. Exclusion criteria: 1) Children with cancer diagnosis; 2) very sick patients. Sample size was computed using the Taro Yamane´s formula for sample size calculation,


$$n=N/1+N{\left(e\right)}^{2}$$

where, n is the desired sample size required, N is the population size which was estimated to be 100, e is the margin of error of 5%. The estimated sample size required (n) was 80 participants. A consecutive sampling approach was utilized to select 80 respondents among the cancer patients who attended the cancer registry within the study time frame. The period for data collection lasted for 3 months (February 2017- April 2017).

**Data collection:** a standardized instrument was used to collect data on quality of life among cancer patients in the study area. For the purpose of the scope of this study, we made use of the standardized Functional Assessment of Cancer Therapy-General (FACT-G). It is a general cancer quality of life instrument for evaluating patients receiving cancer treatment. It consists of 28 items, divided into four subscales for physical, functional, social and emotional well-being [7]. This instrument is considered appropriate for use in any type of cancer. Coefficients of validity and reliability were very high [7]. The scale´s ability to discriminate among patients with different performance status was also significant and, additionally, it has demonstrated sensitivity to change over time [7]. A longitudinal change of 5% in FACT-G score is considered to be clinically meaningful and significant [8]. A validated check list was also adopted to assess various lifestyle modifications of cancer patients receiving varied treatment. This is a 13-item scale that measures overall lifestyle modification of individual with cancer. Participants responded by indicating their agreement to the 13 statements using a four-point scale ranging from “not at all” [1], “a little” [2], “moderate” [3], “very much” [4]. The researchers administered the questionnaires to study participants during their clinic days after gaining their consent. Averagely, participants spent 20 minutes to complete the questionnaires.

**Statistical analysis:** the data collected for the study were first of all checked for errors, cleaned and then analyzed using the Statistical Package for Social Sciences (SPSS), version 20. Descriptive analysis of socio-demographic characteristics of respondents, quality of life of cancer patients, lifestyle modifications etc., were presented in frequencies and percentages using tables. The chi-square test was used to test for significance of association between the independent variables (age, gender, educational level and self- assessment of patient´s health) and dependent variable (quality of life of cancer patients). Multivariate analysis using the logistic regression model was used to determine the factors that significantly predict the quality of life of respondents. Incorporated in the regression model were factors that were significant at the bivariate (chi-square) analysis, although factors that were significant at 0.1 were also included in the multiple regression analysis. The logistic regression was used to adjust for confounding factors.

**Ethical considerations:** ethical approval for this study was obtained from the Ethics and Research Committee of Federal Teaching Hospital, Ido-Ekiti (protocol number: ERC/2017/02/12B). Informed consent was also obtained from the participants before commencement of the study after which questionnaire was distributed to the oncology patients who attended the cancer registry of Federal Teaching Hospital, Ido-Ekiti.

## Results

**General characteristics of study population:** the socio-demographic characteristics of 80 cancer patients recruited for this study are presented in Table 1. Respondent´s ages ranged from 20 - 61 years with a mean age of 50 ± 18.3 years. Higher percentage (31.3%) of the respondents were 61 years and above. Majority of the respondents were Christians (73.8%). Seventy percent of the participants were married. The vast majority (78.8%) of respondents were of Yoruba ethnicity, and 46.3% had tertiary level of education (Table 1). Regarding year of diagnosis, type of treatment received and pain experienced by these patients (Table 2), most of the respondents (43.8%) were diagnosed of having cancer over 2 years ago. Also, a higher percentage (37.5%) started receiving treatment over 2 years ago. Respondents pain experience ranged from mild to moderate to severe pain with moderate bodily pain felt by majority (41.3%) while minority (10%) experienced severe pain and 13% of the patients said they felt no pain at all (Table 2).

**Table 1:** socio-demographic data of respondents

Item	Frequency (n=80)	Percentage (%)
**Age in years**		
20 and below	2	2.5
21- 40	28	35
41- 60	25	31.3
61 and above	25	31.3
**Mean age (SD) = 50 years ± 18.3**		
**Sex**		
Male	28	36.3
Female	51	63.8
**Religion**		
Christianity	59	73.8
Islam	21	26.3
**Marital status**		
Single	12	15.6
Married	58	74.6
Separated	7	9.1
Missing data	3	3.8
**Educational Level**		
Primary	7	8.9
Secondary	23	29.1
Tertiary	37	46.8
No formal education	12	15.2
**Ethnicity**		
Yoruba	63	81.8
Hausa	5	6.5
Igbo	9	11.7
Missing data	3	3.8

**Table 2:** year of diagnosis, treatment modalities, and pain experienced by patients receiving varied cancer treatment at the health facility

Item	Frequency (N=80)	Percentage (%)
**1. When were you diagnosed of having cancer?**		
Less than 6 monthS	12	15.0
One year ago	25	31.3
Over 2 years ago	35	43.8
Over 5 years ago	8	10.0
**2. When did you start receiving treatments?**		
Less than 6 months	19	23.8
One year ago	23	28.8
Over 2 years ago	30	37.5
Over 5 years ago	8	10.0
**3. What type of treatment are you receiving?**		
Chemotherapy	51	63.8
Radiotherapy	9	11.3
Surgical therapy	62	77.5
Hormonal therapy	4	5.0
Others specify	1	1.3
**4. How much bodily pain have you had ever since you started receiving treatment?**		
None	11	13.8
Mild	28	35.0
Moderate	33	41.3
Severe	6	7.5
Very severe	2	2.5

**Quality of life of cancer patients:** the result on quality of life of cancer patients are presented in Table 3, where about 33% of the respondents needs a little bit medical treatment to function in their daily life, 31.6% depends moderately on medication, 11.7% depends extremely on medication and about 7.8% are not dependent at all. Moreover, most of the participants reported that their sex life has been affected (61.1%), few (27.9%) reported difficulty in breathing, about half (45%) reported that their bowel movement has been affected, 39% of the participants experience vomiting and majority said they feel weak (79.7%). Although these symptoms were reported at varying degree.

**Table 3:** quality of life of cancer patients attending Federal Teaching Hospital Ido-Ekiti (FETHI)

Item	Frequency (N=80)	Percentage (%)
**1. Ever since you started receiving treatment, how much did pain interfere with your normal work (including both work outside the home and housework)?**		
Not at all	12	15.4
A little bit	26	33.3
Moderately	27	34.6
Quite a bit	7	9.0
Extremely	5	6.4
Not applicable	1	1.3
**2. How much do you need any medical treatment to function in your daily life?**		
Not at all	9	11.4
A little bit	26	32.9
Moderately	25	31.6
Quite a bit	12	15.2
Extremely	6	7.6
Not applicable	1	1.3
**3. How dependent are you on medication?**		
Not at all	6	7.8
A little bit	26	33.8
Moderately	24	31.2
Quite a bit	11	14.3
Extremely	9	11.7
Not applicable	1	1.3
**4. Has your medical treatment interfered with your sex life?**		
Not at all	15	20.8
A little bit	18	25.0
Moderately	7	9.7
Quite a bit	12	16.7
Extremely	7	9.7
Not applicable	13	18.1
**5. Do you have difficulty in breathing?**		
Not at all	50	63.3
A little bit	10	12.7
Moderately	6	7.6
Quite a bit	4	5.1
Extremely	2	2.5
Not applicable	7	8.9
**6. Is your bowel movement affected?**		
Not at all	39	50.0
A little bit	14	17.9
Moderately	9	11.5
Quite a bit	8	10.3
Extremely	4	5.1
Not applicable	4	5.1
**7. Did you experience any vomiting?**		
Not at all	44	55.7
A little bit	17	21.5
Moderately	6	7.6
Quite a bit	3	3.8
Extremely	5	6.3
Not applicable	4	5.1
**8. Do you feel weak?**		
Not at all	15	19.0
A little bit	25	31.6
Moderately	16	20.3
Quite a bit	11	13.9
Extremely	11	13.9
Not applicable	1	1.3

**Figure 1:**
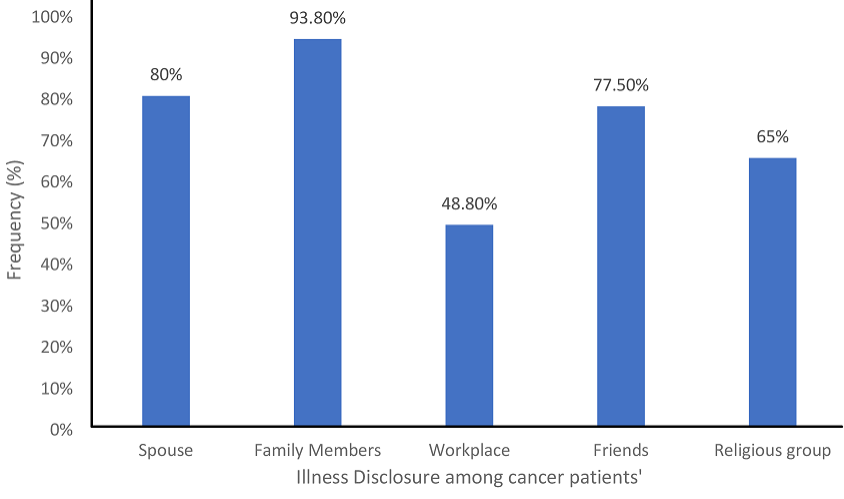
illness disclosure among cancer patients´

From the overall analysis of quality of life (QOL) of respondents, a bit above half (55.1%) of the respondents have good quality of life and about 44.8% have poor quality of life. Regarding illness disclosure to significant others (cancer diagnosis), 80% of the respondent disclosed their illness to their spouse, 93.8% disclosed their illness to their family, 48.8% disclosed it in their work place, 77.5% disclosed it to their friends and about 65% disclosed it to their religious group (Figure 1). However, majority (48.8%) was very much satisfied with the support gotten from spouse, 41.8% was also very much satisfied with the support gotten from the family, whereas minority (5.0%) were satisfied with the support they got from their workplace.

**Lifestyle modifications of cancer patients:** regarding life style modification of cancer patients, a higher percentage (43.8%) are not confident to be able to fulfill their various roles. As touching diet, only 36.3% of the respondents are very much satisfied with their diet. As touching their sleeping pattern, only 29.1% of the respondents are very much satisfied with their sleep pattern and majority (74%) also need assistance to do their day to day activities (Table 4, Table 4 suite).

**Table 4:** respondents response to various lifestyle modifications

Various lifestyle modifications	Frequency (n=80)	Percentage (%)
**Roles** 1. Are you confident that you are able to fulfill your family needs?		
Not at all	10	13.3
A little	41	54.7
Moderate	17	22.7
Very much	7	9.3
Missing data	5	
2. Are you confident that you are able to fulfill your work roles?		
Not at all	10	13.0
A little	35	45.5
Moderate	26	33.5
Very much	6	7.8
Missing data	3	
3. Are you confident that you are able to fulfill society roles?		
Not at all	16	20.3
A little	41	51.9
Moderate	18	22.8
Very much	4	5.1
Missing data	1	
4. Do you feel confident that you are able to manage your financial needs at any situation?		
Not at all	6	7.7
A little	31	39.7
Moderate	34	43.6
Very much	7	9.0
Missing data	2	
5. How satisfied are you with your present sex life?		
Not at all	25	39.7
A little	21	33.3
Moderate	8	12.7
Very much	9	14.3
Missing data	17	
**DIET** 6. Do you feel satisfied with your diet with your present state of appetite?		
Not at all	7	8.8
A little	12	16.3
Moderate	31	38.8
Very much	29	36.3
Moderate	13	16.3
Very much	13	16.3

**Table 4 suite:** respondents response to various lifestyle modifications

Various lifestlye modifications	Frequency (n=80)	Percentage (%)
7. Has anything changed in your diet since you started receiving treatment?		
Not at all	24	30.0
A little	30	37.5
Moderate	13	16.3
Very much	13	16.3
**Activities/exercise**		
8. Do you have enough energy for everyday life?		
Not at all	4	5.3
A little	27	36.0
Moderate	33	44.0
Very much	11	14.7
Missing data	5	
9. Do you need any assistance to do your day-to-day activities		
Not at all	20	25.3
A little	31	39.2
Moderate	19	24.1
Very much	9	11.4
Missing data	1	
10. Did you feel any difficulty walking even a short distance?		
Not at all	33	41.8
A little	18	22.8
Moderate	23	29.1
Very much	5	6.3
Missing data	1	
11. Are you able to walk up and down stairs?		
Not at all	4	5.0
A little	19	24.4
Moderate	37	47.4
Very much	18	23.1
Missing data	2	
**Sleep patterns**		
12. Do you sleep well?		
Not at all	6	7.6
A lttle	15	19.0
Moderate	35	44.3
Very much	23	29.1
Missing data	1	
13. Has this illness affected your sleeping patterns in any way?		
Not at all	29	37.2
A little	22	28.2
Moderate	13	16.7
Very much	14	17.9

**Factors associated with quality of life of cancer patients:** bivariate analysis testing association between factors affecting quality of life of cancer patients revealed there is a significant relationship between the quality of life and self-assessment of health of the patients (p=0.001), although, age (p=0.097), gender (0.184) and respondents education level (0.082) were not significant at bivariate levels (Table 5). Result of multiple regression analysis to determine the factors that significantly predict the quality of life by respondents revealed that self-assessment by respondents (AOR: 3.389; 95% CI: 1.897-6.054) remained a significant and more likely predictor of quality of life while respondent´s age (AOR: 0.244; 95% CI: 0.068-0.876) and level of education (AOR: 0.054; 95% CI: 0.005-0.546) were less likely predictors (Table 6).

**Table 5:** bivariate analysis testing association between age, gender, educational level and self-assessment of patient’s health and quality of life

	Quality of life			
Age group	Poor quality of life	Good quality of life	Total	P value
20 and below	1 (50.0%)	1 (50.0%)	2 (100.0%)	
21-30	6 (66.7%)	3 (33.3%)	9 (100.0%)	0.097
31-40	4 (21.1%)	15 (78.9%)	19 (100.0%)	
41-50	5 (50.0%)	5 (50.0%)	10 (100.0%)	
51-60	9 (64.3%)	5 (35.7%)	14 (100.0%)	
61 and above	10 (40.0%)	15 (60.0%)	25(100.0%)	
Total	35 (44.3%)	44 (55.7%)	79 (100.0%)	
**Educational level**				
No formal education	3 (25.0%)	9 (75.0%)	12 (100.0%)	
Primary	6 (85.7%)	1 (14.3%)	7 (100.0%)	0.082
Secondary	10 (43.5%)	13 (56.5%)	23 (100.0%)	
Tertiary	16 (43.2%)	21 (56.8%)	37 (100.0%)	
**Gender**				
Male	10 (35.7%)	18 (64.3%)	28 (100.0%)	0.184
Female	25 (49.0%)	26 (51.0%)	51 (100.0%)	
**Self-assessment (respondents rating of their own health)**				
Excellent	1 (12.5%)	7 (87.5%)	8 (100.0%)	
Very good	3 (12.5%)	21 (87.5%)	24 (100.0%)	
Good	13 (59.1%)	9 (40.9%)	22 (100.0%)	0.001
Fair	13 (65.0%)	7 (35.0%)	20 (100.0%)	
Poor	5 (100.0%)	0 (0.0%)	5 (100.0%)	
Total	35 (44.3%)	44 (55.7%)	79 (100.0%)	

**Table 6:** predictors of quality of life of respondents

Variables	B	p-value	AOR	95% C.I.	
				Lower	Upper
Assessment	1.221	<0.001*	3.389	1.897	6.054
Age	-1.41	0.03*	0.244	0.068	0.876
Education	-2.927	0.013*	0.054	0.005	0.546

* : P-value < 0.05; B: coefficient of regression; AOR: adjusted odds ratio; 95% CI: 95% confidence interval; predictive value: 74.7%

## Discussion

The aim of this study was to assess the quality of life of cancer patients undergoing varied cancer treatments in a tertiary health institution in Ekiti State and to determine the lifestyle modifications of these patients. The study went further to predict factors associated with quality of life of cancer patients´. In this study, respondent´s ages ranged from 20 - 61 years with a mean age of 50 years. However, there is no significant relationship between the quality of life and age of the patient which is similar to the findings of Lavdaniti *et al*. 2019 [9], where no significant statistical relationship was found between age and physical functioning, bodily pain, general health, vitality, social functioning and mental health except for physical role (p=0.03). Also, it is noteworthy to observe significant number of cancer cases between ages 20-40 years. Although the highest incidence was observe in age group 60 years and above, the proportion (35%) of cases seen in respondents below 40 years of age is however not negligible. This is consistent with the findings of Esan *et al*. 2018 [10], where the most affected group were age group 20-29. This has its own policy implication. This then means that cancer incidences is increasing among the younger age group. This paradigm shift calls for a refocusing of intervention (screening and preventive services) to cut across all age-group.

The present study result showed that 76.3% of cancer patients need varying measure of medical treatment to function in their daily lives and moreover, most of the participants (61.1%) reported that their sex life has been affected. Similar to the findings of Julian *et al*. 2019 [11] that about one third of the patients reported being dissatisfied with their sexuality and having supportive care needs in this area. It is increasingly apparent that these patients experience sexual impairments [12] as well as decreased satisfaction [13] which has significant impact in their psychosocial life. In no doubt, most cancer patients will need their medications and other forms of treatment for optimal functioning for their daily life and must be encouraged and supported to have them. Cancer patients in this series reported varying degree of symptoms with about half (45%) having affectation of bowel movement, 39% experience vomiting and majority said they feel weak (79.7%). This is comparable with the findings of Nayak *et al*. 2017 [14], where most of the participants´ physical well-being was affected by pain 72.9%, sleep problem 71.7%, and fatigue 91.8%.

Regarding support system, most cancer patients have their main support from spouses, 41.8% and family members. Support system is crucial to the improvement on treatment and ultimate survival of cancer patients on medications as Teston *et al*. 2018 [15] discovered that with treatment initiated, participants revealed that absence of a family member/companion in the places treatments were developed awakened loneliness and increased insecurity facing the illness. When treatment plan is being initiated, it is important that the support system for these patients be factored in. The dietary and sleep patterns of cancer patients on treatment in this study were significantly affected and thus up to 43.8% are not confident to be able to fulfill their various roles with only 29.1% having good sleep. This is similar to the findings of Nayak *et al*. 2017 [14], where up to 71.7% also reported disturbed sleep pattern.

Though up to 44.0% of our respondents had enough energy to perform their day to day activities but as much as 74.7% need varying degree assistance to do their day to day activities. This could be because 70.5% and 35.4% had moderate to very much difficulty walking up and down stairs and walking short distance respectively. Coupled with the fact that the mean age of our respondents was 50 years, there could be other health conditions that could affect their day to day activities. Shahidi *et al*. 2014 [16] in their study showed that being diagnosed with and being treated for cancer has a major impact on the day-to-day life of patients with more than 40% reporting changes in at least 8 listed activities after diagnosis with cancer.

This study showed that there is a significant relationship between the quality of life and self-assessment of health of the patients (p=0.001). Araya *et al*. 2020 [17] demonstrated that the global health status/quality of life was significantly associated with emotional functioning (AOR = 5.25, 95%CI=2.2612.17) among other variables. Overall, about 44% have poor quality of life in this current study. This is however in contrast to the findings of Nayak *et al*.2017 [14] where majority (82.3%) of the cancer patients had poor quality. Many of the cancer patients in this study still experience many symptoms that affected their QOL. The findings from other research studies also show that there was a significant reduction in the QOL due to common symptoms resulting from cancer [18,19]. Many authors have reported that side effects of treatment affect the quality of life of cancer patients [20,21]. A lot still need to be done about improving the quality of life of cancer patients in order to ensure maximum productivity even after cancer diagnosis has been made. Cancer management should be geared towards improving/ameliorating symptoms and improving quality of life of cancer patients. One of the limitations of this study was that analyses were based on self-report with the possibility of over and under reporting. It may be difficult to generalize the study findings to cancer patients outside Ekiti State because of cultural diversity and individual perception of health may vary based on location/residence.

## Conclusion

Cancer patients in this study experienced many symptoms in varying degrees that affected their QOL irrespective of their age. Instituting effective support system for cancer patients as part of the management plan is crucial to improving their performance in their day to day activities and ultimately their quality of life while on treatment.

### What is known about this topic

- Cancer is one of the most life threatening illness that may appear at any point in a person´s life and is usually viewed in African context as a death sentence;
- In Nigeria, cancer is seen an as incurable disease and after diagnosis; patients suffer from anxiety and depression resulting from unrealistic fear of death and loss of happiness.

### What this study adds

- Cancer patients receiving treatment experienced many symptoms in varying degrees that affected their QOL irrespective of their age;
- Instituting effective support system for cancer patients as part of the management plan is crucial to improving their performance in their day-to-day activities, thus leading to an improvement in cancer patients´ quality of life.
